# Clusterin facilitates metastasis by EIF3I/Akt/MMP13 signaling in hepatocellular carcinoma

**DOI:** 10.18632/oncotarget.3093

**Published:** 2014-12-30

**Authors:** Cun Wang, Guangzhi Jin, Haojie Jin, Ning Wang, Qin Luo, Yurong Zhang, Dongmei Gao, Kai Jiang, Dishui Gu, Qiujing Shen, Xisong Huo, Fangyuan Hu, Tianxiang Ge, Fangyu Zhao, Wei Chu, Huiqun Shu, Ming Yao, Wenming Cong, Wenxin Qin

**Affiliations:** ^1^ State Key Laboratory of Oncogenes and Related Genes, Shanghai Cancer Institute, Renji Hospital, Shanghai Jiao Tong University School of Medicine, Shanghai, China; ^2^ Department of Pathology, Eastern Hepatobiliary Surgery Hospital, Second Military Medical University, Shanghai, China; ^3^ Liver Cancer Institute, Zhongshan Hospital, Fudan University, Key Laboratory of Carcinogenesis and Cancer Invasion, Ministry of Education, Shanghai, China

**Keywords:** Clusterin, hepatocellular carcinoma, metastasis, prognosis, OGX-011

## Abstract

Clusterin (CLU) is a stress-induced chaperone that confers proliferative and survival advantages to cancer cells. However, effects and molecular mechanisms of CLU in hepatocellular carcinoma (HCC) metastasis are still unknown. In this study, HCC tissue array (n = 198) was utilized to investigate correlation between CLU expression and clinicopathological features. Overexpression of CLU in HCC tissues was correlated with shorter overall survival and higher tumor recurrence. *In vitro* and *in vivo* assays demonstrated that silencing CLU attenuated the invasion and metastasis of HCC cells, whereas ectopic overexpression of CLU resulted in the forced metastasis of HCC cells. We also revealed that CLU activated Akt signaling through complexing with eukaryotic translation initiation factor 3 subunit I (EIF3I), which in turn promoted matrix metalloproteinase 13 (MMP13) expression and HCC metastasis. Positive correlations between CLU and MMP13, p-Akt, or EIF3I were found in HCC tissues. We further observed that CLU knockdown using the CLU inhibitor OGX-011 significantly suppressed HCC metastasis in two metastatic models through inhibiting EIF3I/Akt/MMP13 signaling. These findings indicate that CLU is an independent predictive factor for prognosis of HCC and it facilitates metastasis through EIF3I/Akt/MMP13 signaling. CLU suppression using OGX-011 may represent a promising therapeutic option for suppressing HCC metastasis.

## INTRODUCTION

Hepatocellular carcinoma (HCC) is the fifth most frequent cancer and the third leading cause of cancer-related death worldwide[1, 2]. Although treatment techniques for HCC have experienced great progress, prognosis is still poor for HCC patients due to high rates of tumor recurrence and metastasis[3, 4]. In order to give rise to a metastatic tumor, cancer cells must complete all of the following steps: invasion, intravasation, survival in the circulation, arrest and extravasation into the secondary site. Recently, various proteins and signaling pathways have been found to closely correlate with the recurrence and metastasis of HCC patients[5]. However, molecular mechanisms of HCC metastasis remain poorly understood.

Molecular chaperones are proteins that expressed in response to cellular stresses including genotoxic agents, nutrient starvation, and heat shock. Molecular chaperones assist cells deal with cellular stresses-induced protein misfolding, aggregation, and denaturation. They also play critical roles in cell survival, cell migration, cell adhesion, transformation, and cell-cell interactions[6]. Because of the existence of ischemic and hypoxic microenvironment in cancer tissues, various kinds of molecular chaperones including heat shock protein 27 (Hsp 27)[7], Hsp90[8, 9], αB-crystallin[10], and clusterin (CLU)[11-14] are often adaptively overexpressed and closely related with the increased tumorigenicity, metastatic potential, and resistance to chemotherapy.

CLU, also designated as testosterone-repressed prostate message 2 (TRPM2), apolipoprotein J (APOJ), or sulfated glycoprotein 2 (SGP2), is a heterodimeric glycoprotein that plays important roles in cellular signaling and transcriptional regulatory networks[15, 16]. It has several distinct isoforms with extremely different functions as a result of alternative splicing and post-translational modifications[17]. Secretory clusterin (sCLU) is an endoplasmic reticulum (ER)-targeted 449-amino acid polypeptide that represents the major product of CLU gene. Another isoform of CLU is nuclear CLU (nCLU), which is mainly localized in nucleus. Although mature sCLU is processed through the ER-Golgi secretory pathway, emerging studies reveal that sCLU can also exert its cytoprotective role in cytoplasm[17]. CLU is a stress-induced chaperone that inhibits protein misfolding and aggregation in a manner similar to small HSPs (sHsps) and its promoter region contains an element recognized by HSF-1[18]. As a functional homolog of sHsps, CLU inhibited apoptosis by interacting with activated Bax and protects HCC cells from ER stress-induced apoptosis through a physical interaction with GRP78[12, 19]. Furthermore, CLU facilitated degradation of COMMD1 and I-κB to enhance NF-κB activity in prostate cancer cells[20]. CLU inhibition using its targeted inhibitor (OGX-011) synergistically enhanced the activities of Hsp90 and androgen receptor inhibitor in castrate-resistant prostate cancer[21, 22].

It has been described that CLU also exerts a critical role in cancer metastasis[14, 23]. A positive correlation between the level of CLU and Gleason pattern was observed in prostate cancer. In addition, CLU overexpression promoted invasion and metastasis in renal cancer and HCC[11, 24]. Masaki *et al* demonstrated that CLU could enhance invasive and metastatic abilities of prostate cancer via inducing EMT and suggested that CLU suppression might represent an attractive therapeutic strategy for metastatic progression of prostate cancer[23]. Our previous study indicated that expression level of CLU was significantly higher in metastatic HCC cell lines and cancerous tissues from HCC patients with metastasis. In addition, our findings also suggested that CLU was an important mediator of TGF-β-induced epithelial-mesenchymal transition (EMT)[14]. However, detailed mechanisms of CLU in HCC metastasis are still unclear.

In this study, we first reported that overexpression of CLU was significantly correlated with poor prognosis of HCC patients. Effects of CLU on cell invasiveness and metastasis were investigated by both *in vitro* and *in vivo* assays. Underlying molecular mechanisms of CLU in HCC metastasis were revealed. Furthermore, therapeutic effect of the CLU inhibitor (OGX-011) in suppressing HCC metastasis was also addressed.

## RESULTS

### Upregulation of CLU predicts poor prognosis in HCC patients

**Figure 1:**
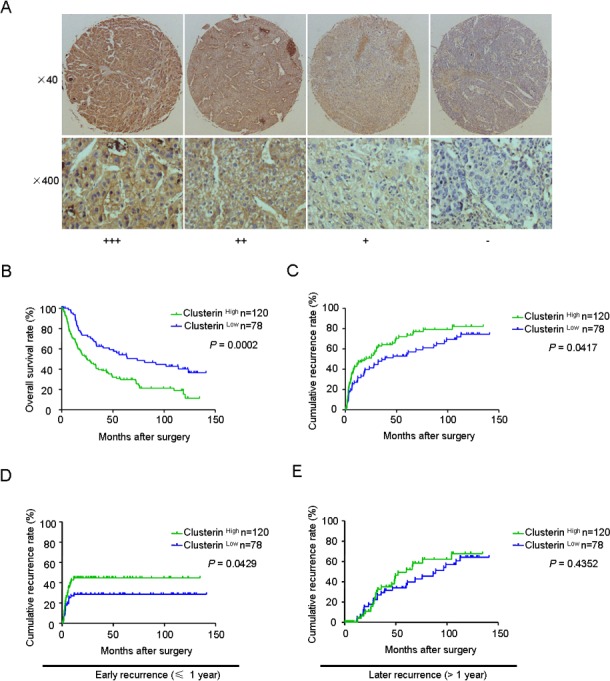
CLU expression correlates with survival and recurrence in HCC patients (A) Typical expressions of CLU in HCC TMAs by immunochemistry analysis (score +++: strong positive; score ++: moderate positive; score +: weak positive; score -: negative); (B and C) Kaplan-Meier analysis of OS and TTR in 198 HCC cases based on CLU expression. (D and E) Prognostic significance of CLU in HCC patients with early recurrence or later recurrence.

We analyzed CLU expression using a tissue microarray (TMA) containing 198 HCC specimens by immunochemistry analysis. CLU protein was predominantly detected in cytoplasm of tumor cells. Levels of CLU protein in tumor tissues were classified as high expression (score ++ and +++) in 120 cases (120/198, 60.6%) and low expression (score +) or not stained (score −) in 78 cases (78/198, 39.4%). Representative images of CLU expression were shown in Figure 1A.

Correlative analysis showed that CLU expression was not correlated with any clinicopathologic features (Supplementary Table 2). However, Kaplan-Meier analysis indicated that mean overall survival (OS) in HCC patients with low expression of CLU was 73.9 months, compared with 24.3 months in HCC patients with high expression of CLU (*P* = 0.0002, log-rank test, Figure 1B). Mean time to recurrence (TTR) in HCC patients with high expression of CLU was 20.4 months, compared with 39.1 months in HCC patients with low expression of CLU (*P* = 0.0417, log-rank test, Figure 1C). In addition, TNM stage, tumor number, tumor differentiation, and microvascular invasion were predictors for OS and/or TTR in univariate analysis (Supplementary Figure 1A-B). Sex, age, hepatitis B virus infection background, AFP, cirrhosis, Child-Pugh score, and tumor size had no prognostic significance for OS and TTR (Table 1). Multivariate Cox regression analysis indicated CLU expression was an independent prognostic factors for postoperative outcome (*P* < 0.001) and tumor recurrence (*P* = 0.014) in HCC patients (Table 1).

**Table 1:** Univariate and multivariate analyses of factors associated with survival and recurrence

	OS				TTR			
		Multivariate				Multivariate		
Factors	Univariate *P*	HR	95% Cl	*P*	Univariate *P*	HR	95% Cl	*P*
Age: ≤ 50 *vs* > 50	0.560	NA	NA	NA	0.401	NA	NA	NA
Sex: male *vs* female	0.284	NA	NA	NA	0.292	NA	NA	NA
HBsAg: negative *vs* positive	0.029	NA	NA	NA	0.153	NA	NA	NA
Serum AFP (ng/ml): ≤ 20 *vs* > 20	0.326	NA	NA	NA	0.747	NA	NA	NA
Liver cirrhosis : no *vs* yes	0.676	NA	NA	NA	0.116	NA	NA	NA
TNM: I *vs* II *vs* III-IV	**0.005**	NA	NA	NA	**0.021**	1.587	1.181-2.132	**0.002**
Child-pugh: A *vs* B	0.255	NA	NA	NA	0.324	NA	NA	NA
Tumor size: ≤ 3 cm *vs* > 3 cm	0.246	NA	NA	NA	0.739	NA	NA	NA
Tumor number: single *vs* multiple	**0.003**	1.843	1.202-2.826	**0.005**	**0.026**	NA	NA	NA
Tumor differentiation: I-II *vs* III-IV	**0.046**	NA	NA	NA	0.567	NA	NA	NA
Microvascular invasion: no *vs* yes	**0.001**	2.346	1.542-3.569	**0.000**	0.143	NA	NA	NA
CLU: high *vs* low	**0.000**	2.354	1.571-3.528	**0.000**	**0.042**	1.628	1.104-2.400	**0.014**

Abbreviations and Note: OS, overall survival; TTR, time to recurrence; NA, not adopted; AFP, alpha-fetoprotein; HBsAg, hepatitis B surface antigen; 95%CI, 95% confidence interval; HR, Hazard ratio. Univariate analysis was calculated by the Cox proportional hazards regression model. Multivariate analysis was done using the Cox multivariate proportional hazard regression model with stepwise manner (forward, likelihood ratio).

We further investigated predictive value of CLU within clinical subgroups (early recurrence and later recurrence). Tumor recurrence was classified as early recurrence and late recurrence using 1 year as the cutoff. According to the time of recurrence, prognostic significance of CLU was existed in the early recurrence group (*P* = 0.0429, Figure 1D) but not in the later recurrence group (*P* = 0.4352, Figure 1E).

### CLU promotes HCC metastasis *in vitro* and *in vivo*

**Figure 2:**
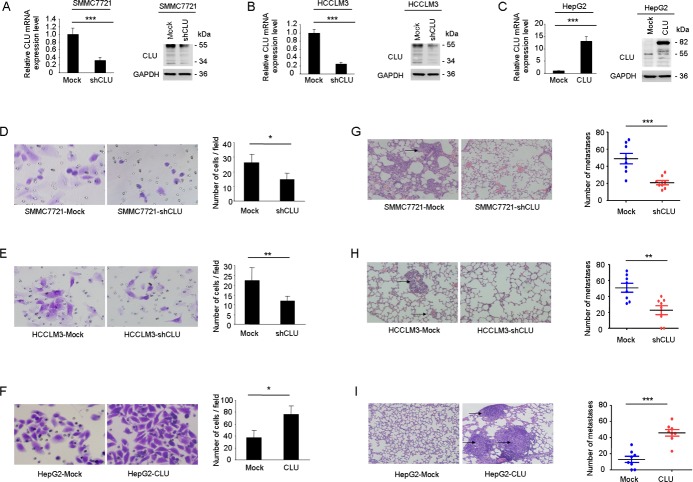
CLU facilitates invasive potential of HCC cells (A and B) qRT-PCR and western blotting were used to validate knockdown of CLU in SMMC7721-shCLU and HCCLM3-shCLU cells. (C) Effective overexpression of CLU in HepG2-CLU cells was confirmed by qRT-PCR and western blot experiments. (D-F) Invasive behavior was analyzed using matrigel invasion assays after knockdown or overexpression of CLU in HCC cells (magnification, ×400). Each experiment was performed in triplicate. (G-I) Representative images of lung tissue sections from each group were shown (magnification, ×200). The number of metastatic nodules in the different groups was also measured. *P < 0.05, **P < 0.01, ***P < 0.001.

In our previous study, we found that CLU expression was positively correlated with metastatic potential of HCC cell lines. Level of CLU expression in SMMC7721 and HCCLM3 was significantly higher compared with HepG2 cells[14]. In order to explore roles of CLU in invasion and metastasis in HCC, we employed lentivirus-mediated shRNA to knockdown CLU in SMMC7721 and HCCLM3 cells and generated a HepG2 cell line ectopically overexpressing CLU. Stable shRNA-mediated knockdown of CLU in SMMC7721 and HCCLM3 cells and effective overexpression of CLU in HepG2 cells were confirmed by qRT-PCR and western blotting (Figure 2A-C). Matrigel invasion assays showed that decreased expression of CLU impaired invasive abilities of SMMC7721 cells (26.4 ± 5.6 vs. 14.8 ± 4.2, *P* < 0.05, Figure 2D) and HCCLM3 cells (22.2 ± 6.8 vs. 12.0 ± 2.3, *P* < 0.01, Figure 2E), whereas invasive potential was significantly enhanced in HepG2-CLU cells compared to mock cells (37.2 ± 12.1 vs. 76.4 ± 14.6, *P* < 0.05, Figure 2F).

Additionally, we established two models of metastatic HCC, including tail vein injected models (SMMC7721 and HepG2) and an orthotopic model (HCCLM3), to investigate roles of CLU in HCC metastasis *in vivo.* Compared to mock cells, CLU knockdown resulted in significant decrease of metastatic foci in SMMC7721 cells (40.9 ± 6.0 vs. 20.9 ± 2.5, *P* = 0.0008, Figure 2G) and HCCLM3 cells (50.8 ± 5.6 vs. 20.6 ± 5.6, *P* = 0.0032, Figure 2H). On the contrary, overexpression of CLU in HepG2 cells increased pulmonary metastases dramatically (12.9 ± 3.8 vs. 45.8 ± 4.1, *P* < 0.001, Figure 2I). Collectively, these results indicate functional significance of CLU expression in HCC metastasis.

### MMP13 is responsible for CLU-mediated HCC invasion

**Figure 3:**
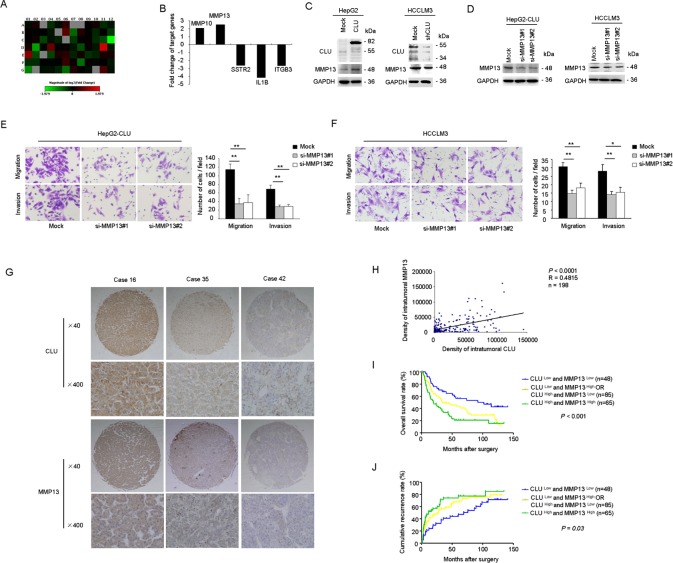
MMP13 plays a crucial role in HCC invasion mediated by CLU (A) Heatmap illustrated alteration of metastasis-related genes in HepG2-CLU cells compared with mock cells using a human tumour metastasis RT^2^ profiler PCR array. (B) Five genes (MMP10, MMP13, SSTR2, IL1B, and ITGB3), which showed a more than 2-fold mRNA differential expression in PCR array, were validated by qRT-PCR. (C) Influence of CLU on MMP13 expression was analyzed by western blotting. (D) Western blotting showed effective knockdown of MMP13 in HepG2-CLU and HCCLM3 cells after siRNA treatment. (E and F) Migration and matrigel invasion assays were done following treatment with MMP13 siRNA (magnification, ×400). Each experiment was performed in triplicate. (G and H) Correlation between CLU expression and MMP13 level was analyzed in tumor tissues derived from 198 patients (Pearson's correlation, r = 0.4815, *P* < 0.0001). Representative images of CLU and MMP13 from three patient samples were shown. (I and J) Co-overexpression of both CLU and MMP13 in HCC patients predicted the worst survival and highest recurrence rate. *P < 0.05, **P < 0.01.

To further investigate molecular mechanisms that CLU involved in HCC invasion, we analyzed metastasis-related genes for HepG2-CLU cells and the relative mock cells using a Tumor Metastasis RT² Profiler PCR Array. The heatmap illustrated relative expression of 84 metastasis-related genes in HepG2-CLU cells, compared with mock cells (Figure 3A). This analysis revealed a total of two upregulated (MMP10 and MMP13) and three downregulated (SSTR2, IL1B, and ITGB3) metastasis-related genes, which had a more than 2-fold change in mRNA levels in HepG2-CLU cells, compared with mock cells. Subsequently, the five candidate targets were validated by qRT-PCR assay (Figure 3B). MMP13 was selected for further investigation because it has the largest upregulated alteration (3.09-fold) in HepG2-CLU cells compared with mock cells. Increased protein level of MMP13 was also observed in HepG2-CLU cells (Figure 3C). On the other hand, after silence of CLU in HCCLM3 cells, MMP13 expression was significantly decreased (Figure 3C). These findings indicate that MMP13 may be regulated by CLU.

To explore whether MMP13 is crucial for CLU-mediated cell invasion, RNA interference was used to silence MMP13 expression in HepG2-CLU and HCCLM3 cells. SiRNA-mediated knockdown of MMP13 in HepG2-CLU and HCCLM3 cells was confirmed using western blotting (Figure 3D). After si-MMP13 treatment, the increased capacities of migration and invasion induced by CLU overexpression were dramatically abolished in HepG2-CLU cells (Figure 3E). Similarly, after MMP13 knockdown in HCCLM3 cells, number of migrative and invasive cells was just approximately 50% of mock cells (Figure 3F). When cells treated with CL82198 (a MMP13 inhibitor), migrative and invasive abilities of HepG2-CLU and HCCLM3 cells were also significantly reduced (Supplementary Figure 2A-B). Taken together, these results reveal that MMP13 plays a crucial role in CLU mediated invasion of HCC.

Additionally, we analyzed MMP13 expression in a TMA containing 198 HCC specimens by immunochemistry analysis. We observed a trend toward better overall survival and lower recurrence rate for patients with lower MMP13 expression (Supplementary Figure 2C-E). Based on our findings, we further tested correlation between expression of CLU and MMP13 in our cohort of HCC tissues. Our results revealed a correlation between expression of CLU and MMP13 (Pearson's correlation, r = 0.4815, *P* < 0.0001, Figure 3G-H). Representative images of CLU and MMP13 from three HCC samples were shown (Figure 3G). Then we divided HCC samples into following three groups: CLU ^Low^ and MMP13 ^Low^, CLU ^Low^/MMP13 ^High^ or CLU ^High^ /MMP13 ^Low^, CLU ^High^ and MMP13 ^High^. We found that 65 of 198 HCC cases (32.8%) exhibited high levels of both CLU and MMP13. HCC patients with high levels of both CLU and MMP13 showed the worst prognoses (*P* < 0.05, Kaplan-Meier analysis, Figure 3I-J). Conversely, HCC patients who expressed low levels of both CLU and MMP13 had better outcomes. These clinical evidences indicate that overexpression of both CLU and MMP13 promotes HCC progression.

### Upregulation of MMP13 by CLU in HCC cells is dependent on Akt activation

**Figure 4:**
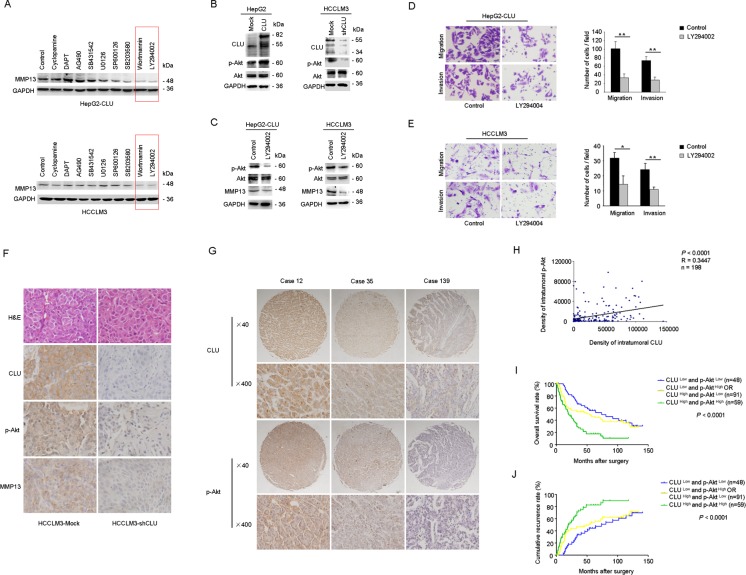
CLU upregulates MMP13 expression through Akt activation (A) HepG2-CLU and HCCLM3 cells were treated with inhibitors of Hedgehog, Notch, Jak, TGF-β, Erk, JNK, p38 kinase, PI3K, or Akt for 24 h. MMP13 expression was detected by western blotting. (B) Effect of CLU on phosphorylation of Akt was analyzed by western blotting. (C) HepG2-CLU and HCCLM3 cells were treated with 50 μM LY294002 for 24 h. Levels of p-Akt, Akt, and MMP13 were detected by western blotting. (D and E) Migration and matrigel invasion assays were done for the HepG2-CLU and HCCLM3 cells following treatment with 50 μM LY294002 for 24 h (magnification, ×400). Each experiment was performed in triplicate. (F) Immunohistochemistry staining of CLU, p-Akt, and MMP13 were performed on serial sections of tumors from HCCLM3-shCLU group and mock group (magnification, ×400). (G and H) Relevance of CLU and p-Akt in HCC tissues was analyzed (Pearson's correlation, r = 0.345, *P* < 0.0001). Representative images of CLU and p-Akt from three patient samples were shown. (I and J) Prognostic values of CLU combined with p-Akt. *P < 0.05, **P < 0.01.

To elucidate which signaling pathway mediates the regulatory effect of CLU on MMP13 expression, HepG2-CLU and HCCLM3 cells were preincubated with inhibitors of Hedgehog, Notch, Jak, TGF-β, Erk, JNK, p38 kinase, PI3K, or Akt for 24 h. Blockade of PI3K-Akt pathway significantly inhibited MMP13 expression in HepG2-CLU and HCCLM3 cells (Figure 4A). Level of p-Akt was significantly higher in HepG2-CLU cells, whereas it was decreased in HCCLM3-shCLU cells, compared with their relative mock cells (Figure 4B). Furthermore, levels of both p-Akt and MMP13 expression were significantly suppressed by LY294002 or si-Akt (Figure 4C and Supplementary Figure 3A). Moreover, inhibition of PI3K-Akt pathway by LY294002 or si-Akt dramatically suppressed migration and invasion of HepG2-CLU and HCCLM3 cells (Figure 4D-E and Supplementary Figure 3B-C). These findings demonstrate that Akt pathway may play a critical regulatory role in MMP13 expression and CLU-induced invasiveness in HCC cells.

The effects of CLU knockdown on levels of MMP13 and p-Akt in orthotopic xenograft tumor tissues were also examined by immunochemistry analysis. Decreased level of CLU accompanied with downregulation of MMP13 and p-Akt was observed in tumors derived from HCCLM3-shCLU group (Figure 4F).

We further analyzed p-Akt level in clinical HCC samples (n=198). The results revealed that p-Akt level was significantly correlated with poor prognosis (Supplementary Figure 3D-F). Moreover, our results indicated a positive correlation between CLU expression and p-Akt level in HCC tissues (Pearson's correlation, r = 0.345, *P* < 0.0001, Figure 4G-H). HCC patients whose tumors with high levels of both CLU and p-Akt exhibited worst prognoses (Kaplan-Meier analysis, *P* < 0.001, Figure 4I-J).

### CLU activates Akt pathway through inhibiting EIF3I degradation

**Figure 5:**
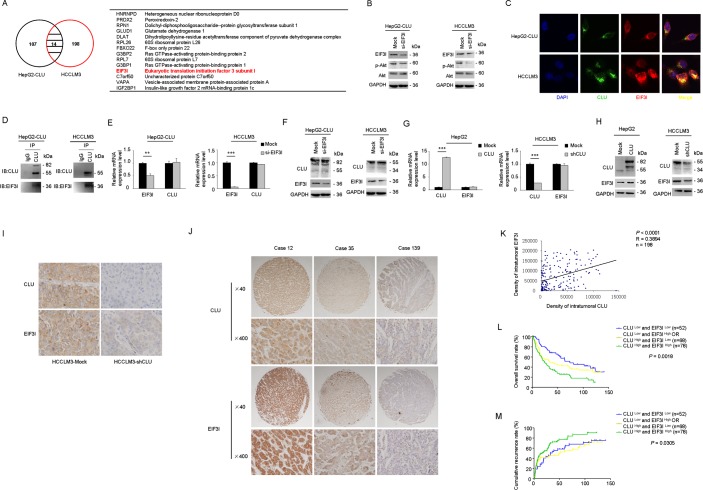
CLU promotes phosphorylation of Akt via interacting with EIF3I (A) Venn diagram showed that a total of 14 proteins were simultaneously identified as CLU-interacting proteins through co-IP and LC-MS/MS in HepG2-CLU and HCCLM3 cells. Overlapping proteins were listed in the table. (B) HepG2-CLU and HCCLM3 cells were transfected with EIF3I siRNA. Levels of EIF3I, p-Akt, and Akt were detected by western blotting. (C) Co-localization of CLU (green) and EIF3I (red) in HepG2-CLU and HCCLM3 cells was validated by immunofluorescence (Original magnification, ×1000). (D) Complex of CLU formed with EIF3I was confirmed by co-IP. (E and F) HepG2-CLU and HCCLM3 cells were transfected with EIF3I siRNA. qRT-PCR and western blotting were employed to detect both mRNA and protein levels of CLU and EIF3I. (G and H) qRT-PCR and western blotting were used to detect both mRNA and protein levels of CLU and EIF3I in HepG2-CLU and HCCLM3-shCLU cells. (I) Immunohistochemistry staining of CLU and EIF3I was performed on serial sections of tumors from HCCLM3-shCLU group and mock group (magnification, ×400). (J and K) Relevance of CLU and EIF3I in HCC tissues (Pearson's correlation, r = 0.3894, *P* < 0.0001). Representative images of CLU and MMP13 from three patient samples were shown. (L and M) Prognostic values of CLU combined with EIF3I. **P < 0.01, ***P < 0.001.

CLU often functions as a cytoprotective molecular chaperone under stress condition. In order to obtain further insight into mechanisms how CLU influences phosphorylation of Akt, a combination of co-IP and LC-MS/MS was used to identify interactome of CLU in HepG2-CLU and HCCLM3 cells. A total of 14 proteins (HNRNPD, PRDX2, RPN1, GLUD1, DLAT, RPL26, FBXO22, G3BP2, RPL7, G3BP1, EIF3I, C7orf50, VAPA, and IGF2BP1) were identified in the two different cell lines (Figure 5A). Of these, EIF3I was found to be related to Akt signaling via WholePathwayScope software analysis and literature retrieval[25]. We found that Akt phosphorylation was significantly inhibited after EIF3I silence (Figure 5B). Confocal microscopy further confirmed co-localization of CLU and EIF3I in cytoplasm in HepG2-CLU and HCCLM3 cells (Figure 5C). Co-IP assay also indicated that CLU formed a complex with EIF3I in HepG2-CLU and HCCLM3 cells (Figure 5D). These results reveal the direct interactions between CLU and EIF3I. More importantly, we found siRNA-mediated inhibition of EIF3I expression had no influence on CLU expression at levels of both protein and mRNA (Figure 5E-F). Up- or down-regulation of CLU expression in HepG2 and HCCLM3 cells caused a corresponding increase or decrease at protein level of EIF3I, respectively, but mRNA level was not changed (Figure 5G-H). These findings suggest that CLU may protect EIF3I protein from degradation. To verify mechanism identified *in vitro*, effects of CLU knockdown on EIF3I expression in orthotopic xenograft tumor tissues were examined by immunochemistry analysis. CLU expression was significantly suppressed in CLU silenced group, which was accompanied by a corresponding downregulation of EIF3I (Figure 5I).

Expression levels of CLU and EIF3I were also analyzed in 198 human HCC tissues using TMAs. Patients with high expression level of EIF3I exhibited poorer prognoses, including time to OS (Supplementary Figure 4A-B, *P* = 0.0573, Kaplan-Meier analysis) and TTR (Supplementary Figure 4C, *P* = 0.0434, Kaplan-Meier analysis). Pearson's correlation analysis indicated that correlation coefficient between CLU and EIF3I was 0.3894 (*P* < 0.0001) (Figure 5J-K). In addition, we found that 78 of 198 HCC cases (39.4%) with high levels of both CLU and EIF3I showed the exacerbated prognosis (Figure 5L-M).

### CLU knockdown using OGX-011 suppresses HCC metastasis

**Figure 6:**
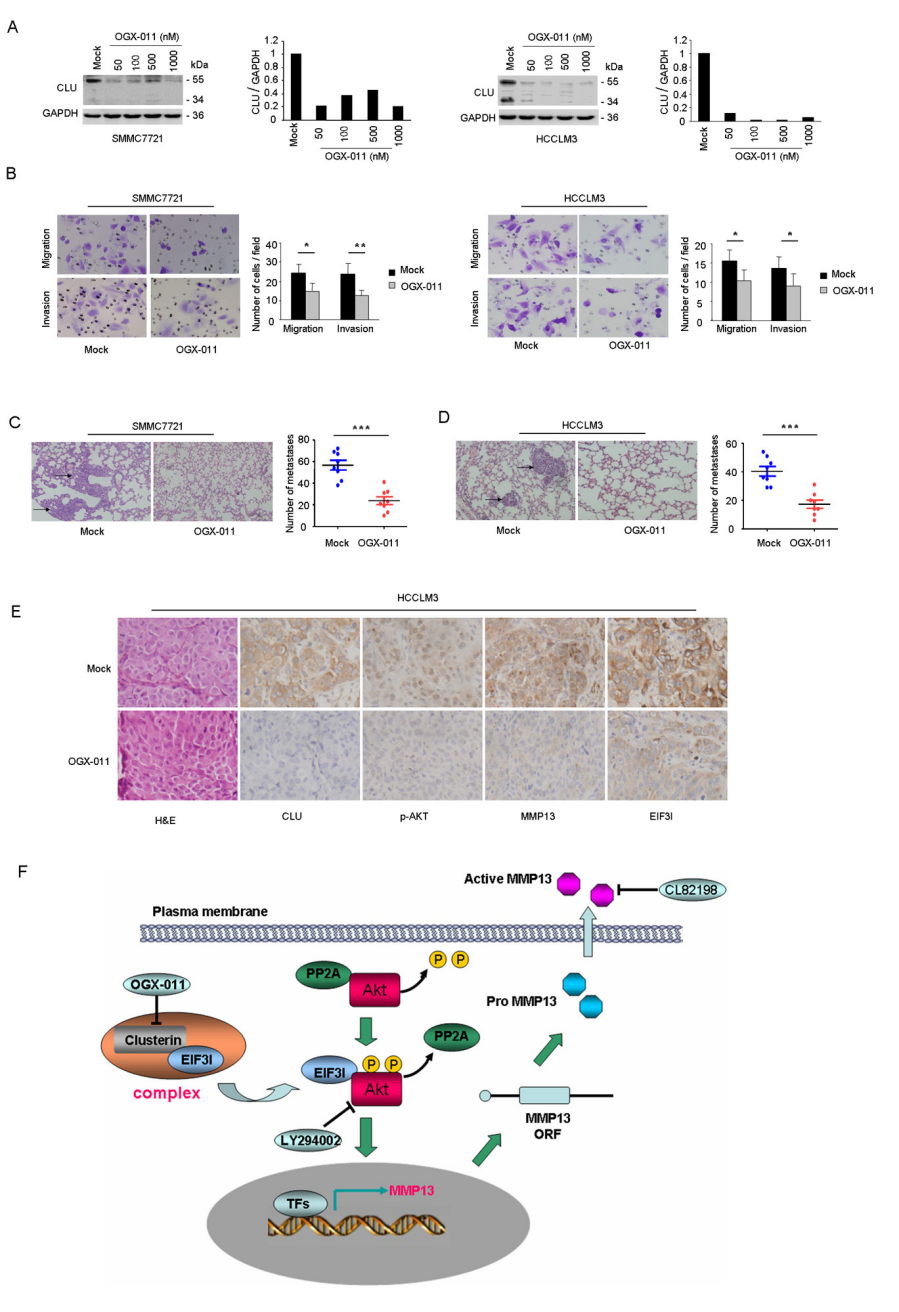
OGX-011 significantly suppresses HCC invasion *in vitro* and *in vivo* (A) SMMC7721 and HCCLM3 cells were treated with different concentration of OGX-011 for 48h. Protein extracts were analyzed for CLU and GAPDH. (B and C) Results of migration and matrigel invasion assays showed the inhibitory roles of OGX-011 on SMMC7721 and HCCLM3 cells. Each experiment was performed in triplicate. (D and E) Lung metastasis from mice treated with OGX-011 or control oligodeoxynucleotide were measured through H&E staining in both tail vein injected model (SMMC7721) and orthotopic model (HCCLM3). Characteristic images of lung metastases by H&E staining were shown. The number of lung metastatic nodules was quantified on serial sections of H&E staining (magnification, ×200). (F) H&E staining and immunohistochemistry staining with anti-CLU, anti-p-Akt, anti-MMP13, and anti-EIF3I were performed on serial sections of tumor specimen (magnification, ×400). (G) Model of CLU-mediated invasion and metastasis was illustrated in HCC. *P < 0.05, **P < 0.01, ***P < 0.001.

Based on the above findings, we next observed effects of CLU suppression on HCC invasive and metastatic ability *in vitro* and *in vivo* using a CLU inhibitor, OGX-011. Western blot analysis was utilized to evaluate suppressive effect of OGX-011 on CLU expression in SMMC7721, HCCLM3, MHCC97L, and MHCC97H cells. OGX-011 significantly suppressed CLU expression in SMMC7721, HCCLM3, MHCC97L, and MHCC97H cells at the concentration of 50 nM (Figure 6A and Supplementary Figure 5A). Moreover, OGX-011 (100 nM) significantly impaired migrative and invasive ability of SMMC7721, HCCLM3, MHCC97L, and MHCC97H cells (Figure 6B, Figure S5B). In order to investigate the inhibitory roles of OGX-011 on HCC metastatic ability *in vivo*, we established two metastatic models including a tail vein injected model (SMMC7721) and an orthotopic model (HCCLM3) as described in method. Mice were treated intraperitoneally with 12.5 mg/kg of OGX-011 once daily for 7 days followed by three weekly treatments thereafter. Lung metastasis was determined by examining serial sections of every lung tissue block by microscopy. Results indicated that OGX-011 significantly reduced number of metastatic nodules with 2.38-fold decrease in the tail vein injected model (*P* < 0.001, Figure 6C) and 2.34-fold decrease in the orthotopic model (*P* < 0.001, Figure 6D). Immunochemistry analysis also revealed that tumors which treated by OGX-011 exhibited the downregulation of CLU and its downstream targets compared with control group (Figure 6E). These *in vivo* evidences suggest that OGX-011 significantly inhibit HCC metastasis.

## DISCUSSION

Recurrence and metastasis are the most leading causes of poor prognosis of HCC patients. Although great efforts have been made to explore molecular mechanism of invasion and metastasis for HCC in the past decade, metastatic mechanism of HCC remains incompletely understood. Growing evidences indicate molecular chaperones, including heat-shock proteins (Hsps), play an important role in cancer metastasis[6]. In this study, we presented the first evidence that CLU, as a functional homolog of Hsps, was closely associated with poor prognosis of HCC patients. A positive correlation between expression level of CLU and early HCC recurrence was found and suggested that cancer cells with high expression of CLU have more invasive phenotype (Figure 1). These results indicated that CLU might be a prospective prognostic marker for HCC patients. Based on depletion or overexpression experiments, we found that overexpression of CLU significantly promoted invasion of HCC cells *in vitro* and contributed to distant lung metastasis *in vivo*. In contrast, silencing of CLU decreased the invasive ability of HCC cells *in vitro* and *in vivo* (Figure 2). It has been reported that CLU overexpression can enhance metastatic potential in prostate cancer[23], renal cell carcinoma[24], and breast cancer[26]. Our above findings link CLU overexpression to cancer metastasis and indicate that CLU may play a crucial role in HCC metastasis.

To investigate the potential molecular mechanism involving CLU and HCC invasiveness, we profiled differentially expressed metastasis-related genes between mock and HepG2-CLU cells using a Tumor Metastasis PCR array. MMP13 was identified as a candidate target regulated by CLU. Expression level of MMP13 was found to be down-regulated under condition of CLU knockdown, while overexpression of CLU resulted in upregulation of MMP13 (Figure 3C). Depletion or inactivation of MMP13 significantly inhibited CLU-mediated invasion of HCC cells (Figure 3E-F). MMP13 is produced by various types of cancer cells and degrades collagen types I, II and III. It is an important regulator of metastatic process in human cancers[27-29]. Increased expression of MMP13 in oesophageal cancer is related to cancer aggressiveness[30]. Tumor-derived MMP-13 correlates with aggressive tumor phenotypes, and inversely correlated with the overall survival of breast cancer patients[31]. In our present study, overexpression of MMP13 was not only closely related with poor prognosis of HCC patients, but also positively correlated with CLU expression in our cohort of HCC tissues (Figure 3G-H). Furthermore, combination of CLU and MMP13 was a more powerful prognostic marker for patients with HCC. These data suggest that MMP13 is involved in CLU-induced invasiveness in HCC cells and may be a critical downstream target of CLU.

We further screened signal pathways involved in regulation of MMP13 expression by treating HCC cells with various small chemical inhibitors against key factors of different signaling pathways. We found that blockade of PI3K-Akt pathway significantly inhibited MMP13 expression in HepG2-CLU and HCCLM3 cells (Figure 4A). Previous study revealed that CLU promoted cell survival through the PI3K/Akt pathway[32]. And CLU induced matrix metalloproteinase-9 expression via ERK1/2 and PI3K/Akt/NF-κB pathways in monocytes/macrophages[33]. In our study, we observed that overexpression of CLU could increase levels of p-Akt and MMP13 expression, whereas CLU knockdown decreased the levels of p-Akt and MMP13. These *in vitro* findings were further validated by clinical samples. A positive correlation between CLU expression and p-Akt level was observed in our cohort of HCC tissues (Figure 4G-H). Taken together, these results show that CLU can promote HCC metastasis through Akt-MMP13 signaling.

Based on results of co-IP and LC-MS/MS analysis, we established interactome of CLU in HCC cells. Western blotting and immunofluorescence analysis further confirmed that CLU was physically associated with EIF3I. In addition, our results indicated that overexpression of CLU was accompanied with up-regulation in level of EIF3I protein, but not EIF3I mRNA in HCC cells (Figure 5E-H). These findings suggest that CLU may protect EIF3I protein from degradation. Next, we observed a positive correlation between CLU and EIF3I in HCC samples, supporting the notion that CLU-EIF3I complex may function as a cooperative unit in cancer cells. EIF3I (also known as eIF3S2) was initially identified as a TGF-β receptor interacting protein and functions as a negative regulator for the TGF-β signaling pathway. EIF3I overexpression was observed in many kinds of cancers, including HCC[25, 34, 35]. In our study, we found that Akt phosphorylation was significantly inhibited when EIF3I was silenced. Wang *et al* also reported that EIF3I could complex with Akt and prevent PP2A-mediated dephosphorylation, which in turn leading to a constitutive activation of Akt signaling [25]. These results suggest that CLU complex with EIF3I and prevent its degradation, then leading to up-regulation of Akt activity.

Multiple studies supported that CLU inhibition might represent a promising therapeutic option for suppressing metastatic progression of prostate cancer[22, 36]. Therefore, we further analyzed the effects of CLU suppression on HCC invasive and metastatic ability using the CLU inhibitor OGX-011. Our results showed that OGX-011 significantly reduced number of lung metastatic nodules in both tail vein injected model and orthotopic model (Figure 6C-D). These results indicate that CLU is a potential therapeutic target for HCC metastasis.

In conclusion, our findings unravel a novel mechanism that CLU complexes with EIF3I and activates Akt pathway, which in turn promotes expression of MMP13 and leads to facilitated metastasis of HCC cells (Figure 6F). These data also support that targeting CLU may be a rational strategy for suppressing metastasis in HCC.

## MATERIALS AND METHODS

### Cell culture

The human HCC cell line HepG2 was purchased from the American Type Culture Collection (ATCC, VA, USA). HCC cell line SMMC7721 was purchased from Shanghai Institute of Cell Biology, Chinese Academy of Sciences. HCC cell lines with stepwise metastatic potential (MHCC97L, MHCC97H and HCCLM3) which are HBV-positive cell lines with the same genetic background but different lung metastatic potentials were established by Liver Cancer Institute of Fudan University. All the cell lines were cultured in Dulbecco's Modified Eagle's Medium (DMEM) supplemented with 10% fetal bovine serum (FBS) and antibiotics (100 U/ml penicillin, 100 mg/ml streptomycin), in a 5% CO_2_ atmosphere at 37°C. For treatment with Cyclopamine (Hh pathway-related protein SMO inhibitor), DAPT (Beta Amyloid inhibitor), AG490 (JAK2 inhibitor), SB431542 (TGFβ1 receptor inhibitor), U0126 (MEK inhibitor), SP6001126 (JNK1/2/3 inhibitor), SB203580 (p38 inhibitor), Wortmannin / LY294002 (PI3K-Akt inhibitor), or CL82198 (MMP13 activity inhibitor), cells were cultured at dose of 10 μM, 10 μM, 20 μM, 10 μM, 1μM, 5μM, 10μM, 100μM / 50μM, or 10μg/ml, respectively, according to the manufacturer's instructions.

### Clinical samples and immunohistochemistry staining

Tumor specimens used in tissue microarrays (TMAs) were obtained from 198 HCC patients who underwent curative resection in Eastern Hepatobiliary Hospital of the Second Military Medical University between May 2003 to September 2006. Patients were selected on the basis of the following inclusion and exclusion criteria: (i) definite pathological diagnosis of hepatocellular lesions according to the World Health Organization criteria; (ii) suitable formalin-fixed, paraffin-embedded tissue; (iii) without pre-operative anti-cancer treatment and no evidence of extrahepatic metastases; (iv) complete clinicopathologic and follow-up data for the patients. Ethical approval was obtained from the Eastern Hepatobiliary Hospital Research Ethics Committee, and written informed consent was obtained from each patient. The overall survival (OS) was defined as the length of time between the surgery and death. For surviving patients, the data were censored at the last follow-up. The time to recurrence (TTR) was defined as the length of time between the date of the surgery and the date of detection of any type of tumor recurrence (intrahepatic recurrence and extrahepatic metastasis).

TMAs were constructed by Shanghai Biochip Co, Ltd, Shanghai, China, as previously described. Immunohistochemistry was performed and integrated optical density (IOD) was measured as reported previously[37]. Antibodies used for immunohistochemical staining included antibody against CLU (sc-6420; Santa Cruz Biotechnology; 1:200 dilution), antibody against MMP13 (sc-30073; Santa Cruz Biotechnology; 1:200 dilution), antibody against p-Akt (2118-1, Epitomics; 1:100 dilution), antibody against EIF3I (ab40745; Abcam; 1:200 dilution). Immunohistochemical score was independently assessed by 2 pathologists without knowledge of patient characteristics.

### Lentivirus production and transduction

Lentivirus production and transduction were performed as previously described[14]. The experimental procedures are described in detail in the Supplementary Materials and Methods.

### RNA isolation and real-time RT-PCR

RNA isolation and real-time RT-PCR were performed as previously described[38]. Primers used were listed in Supplementary Table 1. The experimental procedures are described in detail in the Supplementary Materials and Methods.

### Protein extraction and western blotting

Protein extraction and western blotting were performed as previously described[14]. Cell lysates were extracted with the T-PER tissue protein extraction reagent (Pierce, Rockford, IL) with a cocktail of proteinase inhibitors (Roche Applied Science, Switzerland) and a cocktail of phosphatase inhibitors (Roche Applied Science). Equal amounts of total proteins (20 μg) were separated by 10% SDS-PAGE and transferred onto PVDF membrane using a Bio-Rad SemiDry apparatus. After blocking for nonspecific binding, the membranes were incubated with anti-CLU (1:200 dilution; Santa Cruz Biotechnology), MMP13 (1:200 dilution; Santa Cruz Biotechnology), p-Akt (1:1000 dilution; Cell Signaling Technology), Akt (1:1000 dilution; Cell Signaling Technology), EIF3I (1:800 dilution; Abcam, Hong Kong, China) or GAPDH (1:5000 dilution; Kang-Chen, Shanghai, China) overnight at 4°C, followed by HRP-conjugated secondary antibodies for 1 h at room temperature. After washing three times in TBST, protein bands were visualized using chemiluminescence detection.

### Tumor metastasis PCR array analysis

The Human Tumor Metastasis RT² Profiler™ PCR Array, which is designed to represent 84 genes known to be involved in metastasis, was used to profile mock and CLU-HepG2 cells according to the manufacturer's instructions. Briefly, total RNA was extracted and reverse transcribed into cDNA using an RT^2^ First Strand Kit (Qiagen). Then, combine the template with an instrument specific and ready-to-use RT^2^ SYBR Green qPCR Master Mix. Add equal aliquots of this mixture (25 μl) to each well of the same PCR Array plate containing the predispensed gene-specific primer sets. Real-time PCR and data collection were performed on Applied Biosystems® 7500 Real-Time PCR Systems.

### Gene knockdown using siRNA

SiRNA, including control, MMP13, Akt and EIF3I were all purchased from Biotend (Shanghai, China). The experimental procedures are described in detail in the Supplementary Materials and Methods.

### Migration and invasion assays

See Supplementary Materials and Methods.

### *In vivo* metastasis assays

Two metastatic cancer models including tail vein injected models (SMMC7721 and HepG2) and an orthotopic model (HCCLM3) were established to validate the role of CLU on HCC metastatic ability *in vivo*.

For the tail vein metastasis models, 2×10^6^ cells (SMMC7721 and HepG2) suspended in 200μl serum-free DMEM were injected into the tail vein of nude mice. After six weeks, all of mice were sacrificed. The lung tissues were dissected and fixed with 10% formalin for at least 72 h. Lung tissues were analyzed by hematoxylin and eosin staining.

For the orthotopic metastasis model, HCCLM3 cells infected with lentivirus (CLU-shRNA HCCLM3 and mock cells) were implanted subcutaneously into the upper left flank region of nude mice. When the tumors reached 1 cm in diameter, they were cut into 2 × 2 × 2 mm^3^ sized pieces, and implanted into livers of 8 nude mice, respectively. After 6 weeks, the mice were sacrificed and lungs were fixed with 10% buffered formalin and embedded with paraffin. Lung metastasis was determined by examining serial sections of every lung tissue block by microscopy.

### Co-Immunoprecipitation (Co-IP)

For Co-IP, pre-cleared protein from whole cell lysates were incubated with goat anti-CLU antibody which is conjugated to AminoLink Plus Resin (Pierce) overnight at 4 °C. The IP targets were disassociated from the immobilized antibodies on the AminoLink Plus Resin by the gentle (non-reducing, non-denaturing) elution buffer. Eluted proteins were resolved using 10% SDS-PAGE, followed by western blot with appropriate antibodies and detection using chemiluminescence detection.

### In-gel tryptic digestion and two-dimensional liquid chromatography coupled with tandem mass spectrometry (2D-LC-MS/MS)

Co-IP was performed as described above. Eluted proteins were resolved on an SDS-PAGE denaturing gel, visualized by coomassie blue staining, and the protein band of interest was removed for MS analysis. MS was performed under 19-Kv accelerating voltage in reflection mode with an m/z range of 400 to 2,000. All MS/MS data were identified using SEQUEST (v.28, Bioworks 3.3 software package, Thermo Electron) against the Human International Protein Index (IPI) database.

### Immunofluorescence and confocal microscopy

See Supplementary Materials and Methods for details.

### OGX-011 treatment

OGX-011 is a second-generation 21-mer ODN with a 2′-O-(2-methoxy) ethyl modification, generously provided by Teva Pharmaceutical Industries. The sequence of the OGX-011 was 5′-CAGCAGCAGAGTCTTCATCAT-3′, which targeted the translation initiation site of human CLU gene. The sequence of ODN used as a control in this study was 5′-CAGCGCTGACAACAGTTTCAT-3′.

Two metastatic cancer models including a tail vein injected model (SMMC7721) and an orthotopic model (HCCLM3) were established to validate the effects of OGX-011 on HCC metastatic ability *in vivo*. Each experimental group consisted of 8 mice. After randomization, 12.5 mg/kg of control ODN or OGX-011 was injected i.p. once daily for 7 days followed by three weekly treatments thereafter. After 6 weeks, the mice were sacrificed and lungs were fixed with 10% buffered formalin and embedded with paraffin. Lung metastasis was determined by examining serial sections of every lung tissue block by microscopy.

### Statistical analysis

Statistical analysis was performed with SPSS 15.0 for Windows (SPSS, Chicago, IL). Quantitative variables were analyzed by Student *t* tests. Univariate and multivariate analyses were based on a Cox regression model. The relationship between the expressions of the biomarkers was analyzed by calculating Spearman's correlation coefficient. *P* < 0.05 was considered statistically significant.

## SUPPLEMENTARY MATERIALS AND METHODS


