# Supplementary material for: Clusterin facilitates metastasis by EIF3I/Akt/MMP13 signaling in hepatocellular carcinoma
Source: Oncotarget. 2014 Dec 30;6(5):2903–16. doi: 10.18632/oncotarget.3093 (PMC4413626; doi:10.18632/oncotarget.3093)
Supplement: Supplementary file 1 [file oncotarget-06-2903-s001.pdf]

Clusterin facilitates metastasis by EIF3I/Akt/MMP13 signaling in hepatocellular carcinoma

SUPPLEMENTARY MATERIALS AND METHODS

Lentivirus production and transduction

pGCSIL-CLU-shRNA, a lentiviral vector of CLU-RNA interference was constructed by Shanghai GeneChem Co, Ltd. A GFP-lentiviral vector (pGCSIL-GFP) was used as a negative control. A double-stranded oligonucleotide specific to exon II of the sCLU mRNA (CCGGGATGAAGACTCTGCTGCTGTTCTCGAGAACAGCAGCAGAGTCT TCATCTTTTTG) was annealed and inserted into the shRNA expression vector pGCSIL-GFP. Because the exon II is unique to sCLU, the shRNAs target exon II is specific to sCLU and have no effects on other forms CLU. Lentiviral carrying CLU shRNA or scrambled shRNA were used to infect target cells (SMMC7721 and HCCLM3). The lentivirus was transfected into SMMC7721 and HCCLM3 cells with a multiplicity of infection (MOI) of 10 to 50 (optimal MOI is 20). All lentiviral vectors expressed GFP, which allowed us to measure their infection efficiency in transfected cells.

The cDNA encoding CLU was amplified by reverse transcription polymerase chain reaction (RT-PCR). The cDNA of CLU was cloned into pGC-FU vector. Lentiviral carrying CLU cDNA were generated and harvested as described previously. Briefly, HepG2 cells were infected twice for a total of 2 days (1 day for each infection). Control cell line was generated by infection with viruses containing the empty vector by following the same protocol.

RNA isolation and real-time RT-PCR

Total RNA was extracted from cells using TRIzol reagent (Invitrogen) and reversely transcribed using the PrimeScript™ RT Reagent Kit (Perfect Real Time) (TaKaRa Biotechnology). The real-time polymerase chain reaction (PCR) was subsequently performed according to the manufacturer's instructions (TaKaRa Biotechnology). The expression levels were normalized against the internal reference gene GAPDH, and the relative expression levels were displayed using the $2^{-\Delta\Delta Ct}$ method.

Gene knockdown using siRNA

Short interfering RNA specifically against MMP13, Akt and EIF3I genes (Biotend, Shanghai, China) were transfected into HCC cells using Lipofectamine 2000 reagent (Invitrogen, Carlsbad, CA, USA) according to the manufacturer's instructions. The siRNA sequence targeting human Akt was as follows: CUGACCAAGAUGACAGCAU. The siRNA sequences targeting human MMP13 were as follows: (I) UCUGAACUGGGUCUUCCAA; (II) UCUGAACUGGGUCUUCCAA. The siRNA sequence targeting human EIF3I was GAUCAACGACAUCCAGUUA. The silencing effect was measured by western blotting 72 h post-transfection.

Migration and invasion assays

Cell migration was performed by transwell assay (BD Biosciences, CA, USA). Briefly, 5×10^4 cells in serum-free DMEM were seeded on a membrane (8.0-μm pore size) inserted in a well of a 24-well plate. DMEM containing 10% FBS was added to the lower chamber of each well. After 24 h, cells in the upper chamber were removed by cotton swab and the cells that had reached the underside of the membrane were fixed and stained with crystal violet (1% in methyl alcohol) for 10 min. The cells that located on

the underside of the filter (5 fields / filter) were counted. The cell invasion assay was carried out similarly, except that the matrigel (BD Biosciences, CA, USA) was added to each well 6 h before cells were seeded on the membrane. After 48 h, matrigel and any remaining cells in the upper chamber were removed by cotton swabs. Cells on the lower surface of the membrane were fixed and stained as described above.

Immunocytochemistry and confocal microscopy

Cells were grown on glass coverslips. After an attachment period of 24 h, cells were fixed in 4% formaldehyde at room temperature for 30 min and permeabilized with 0.1% Triton-X in PBS for 10 min and blocked with 10% donkey serum in PBS for 1 h. The coverslips were then incubated with rabbit anti-EIF3I antibody for 1 h, followed by rhodamine-conjugated donkey anti-rabbit IgG for 30 min. Subsequently, goat anti-CLU antibody was applied for another hour, and then stained with FITC-conjugated donkey anti-goat IgG for 30 min. Finally, cells were washed and stained with DAPI. The signals were detected with A1R MP Multiphoton Confocal Microscope and analyzed with NIS-elements microscope imaging software (Nikon Instruments Inc, Melville, NY, USA).

Supplementary Table 1: The sequence of primers used for real-time PCR

Gene name	Forward (5'-3')	Reverse (5'-3')
CLU	GAGCAGCTGAACGAGCAGTTT	CTTCGCCTTGCGTGAGGT
MMP10	AAGGTAAGGGCAGTGAGAATGA	CTTTTGCTGCCCCACTCAGA
MMP13	TGATGATGATGAAACCTGGACA	AGACCTAAGGAGTGGCCGAAC
SSTR2	CTCAAACCAGACAGAGCCGT	TTGGCATAGCGGAGGATGAC
IL1B	GACCACCACTACAGCAAGGG	GTGCATCGTGCACATAAGCC
ITGB3	ACCAGTAACCTGCGGATTGG	CTCATTGAAGCGGGTCACCT
EIF3I	CGGGATGAAGCCGATCCTAC	GGGTCCTTGGCCACAGTAAA

Supplementary Table 2: Correlation between CLU and clinicopathological characteristics

Variable (missing caces)	All cases	CLU expression		*P* value
		Negative expression	Positive expression	
Age				0.771
≤ 50	99	40	59	
> 50	99	38	61	
Sex				0.628
male	167	67	100	
female	31	11	20	
HBsAg				0.525
negative	51	22	29	
positive	147	56	91	
Serum AFP				0.201
≤ 20 ng/ml	73	33	40	
> 20ng/ml	125	45	80	
Liver cirrhosis				0.588
no	20	9	11	
yes	178	69	109	
TNM				0.244
I	57	20	37	
II	116	51	65	
III-IV	25	7	18	
Child-pugh class				0.703
A	189	75	114	
B	9	3	6	
Tumor size				0.517
≤ 3 cm	24	8	16	
> 3 cm	174	70	104	
Tumor number				0.440
single	157	64	93	
multiple	41	14	27	
Tumor differentiation (3)				0.937
I-II	32	13	19	
III-IV	163	65	98	
Vascular invasion				0.758
no	66	25	41	
yes	132	53	79	

Abbreviations: AFP, alpha-fetoprotein; HBsAg, hepatitis B surface antigen; TNM, tumor-node-metastasis.

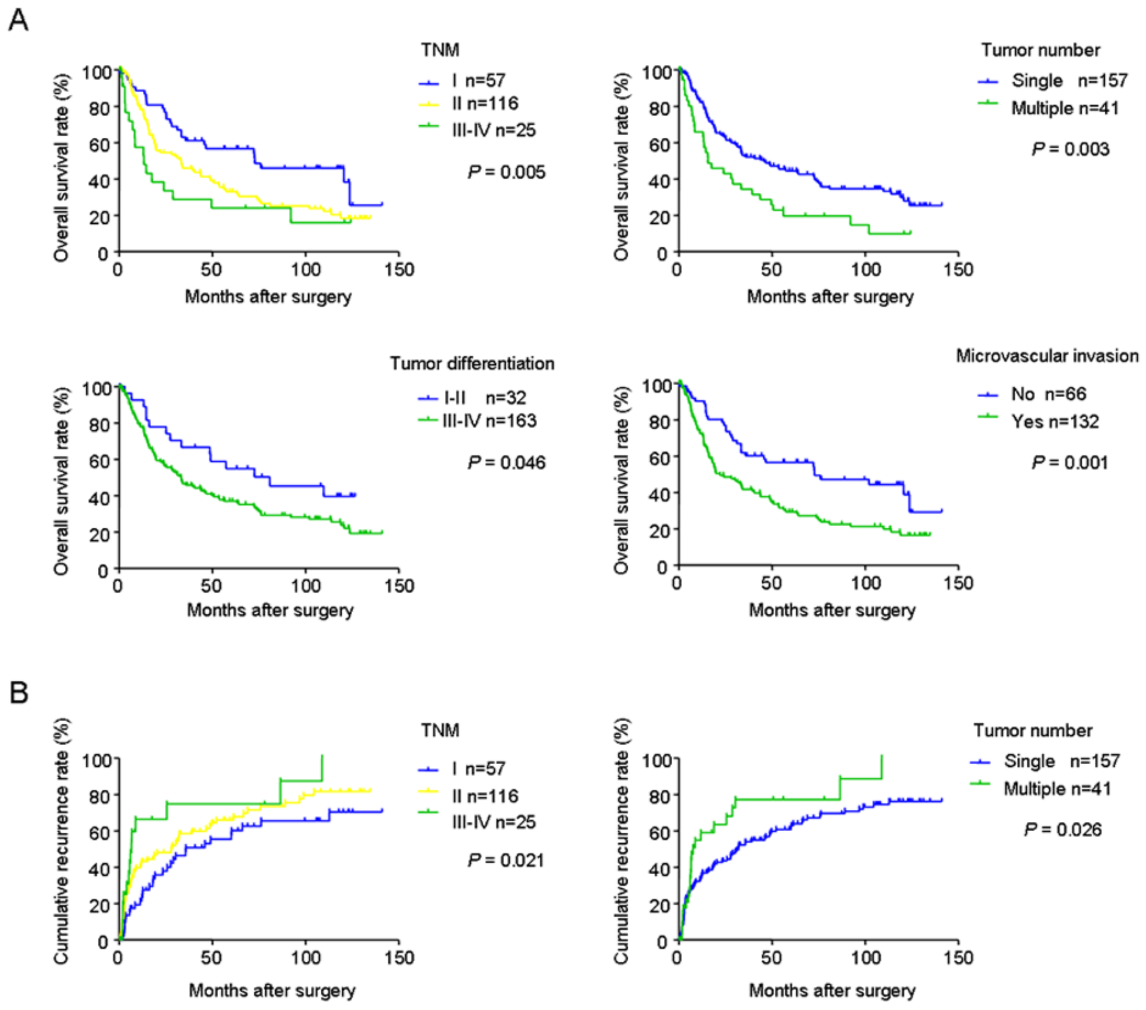

Supplementary Figure 1: Kaplan-Meier curves of OS and / or TTR for TNM classification, tumor number, tumor differentiation, and microvascular invasion among HCC patients.

(A) TNM classification, tumor number, tumor differentiation, and microvascular invasion were found to be significantly associated with OS. (B) TNM classification and tumor number were found to be significantly associated with TTR.

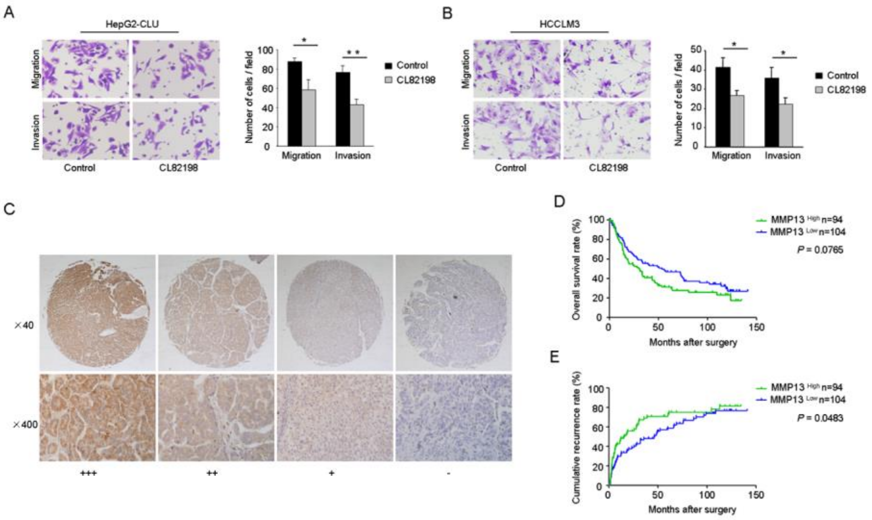

Supplementary Figure 2: Suppressive effect of MMP13 inhibitor CL82198 on migration and invasion of HepG2-CLU and HCCLM3 cells.

(A and B) Migration and matrigel invasion assays were done for the HepG2-CLU and HCCLM3 cells following treatment with CL82198 (10 µg/ml) for 24 h (magnification, ×400). Each experiment was performed in triplicate. (C) Typical expression of MMP13 in HCC tissue microarrays by immunochemistry analysis (score +++: strong positive; score ++: moderate positive; score +: weak positive; score -: negative); (D and E) Kaplan-Meier analysis of OS and TTR in 198 HCC cases based on MMP13 expression. *P < 0.05, **P < 0.01.

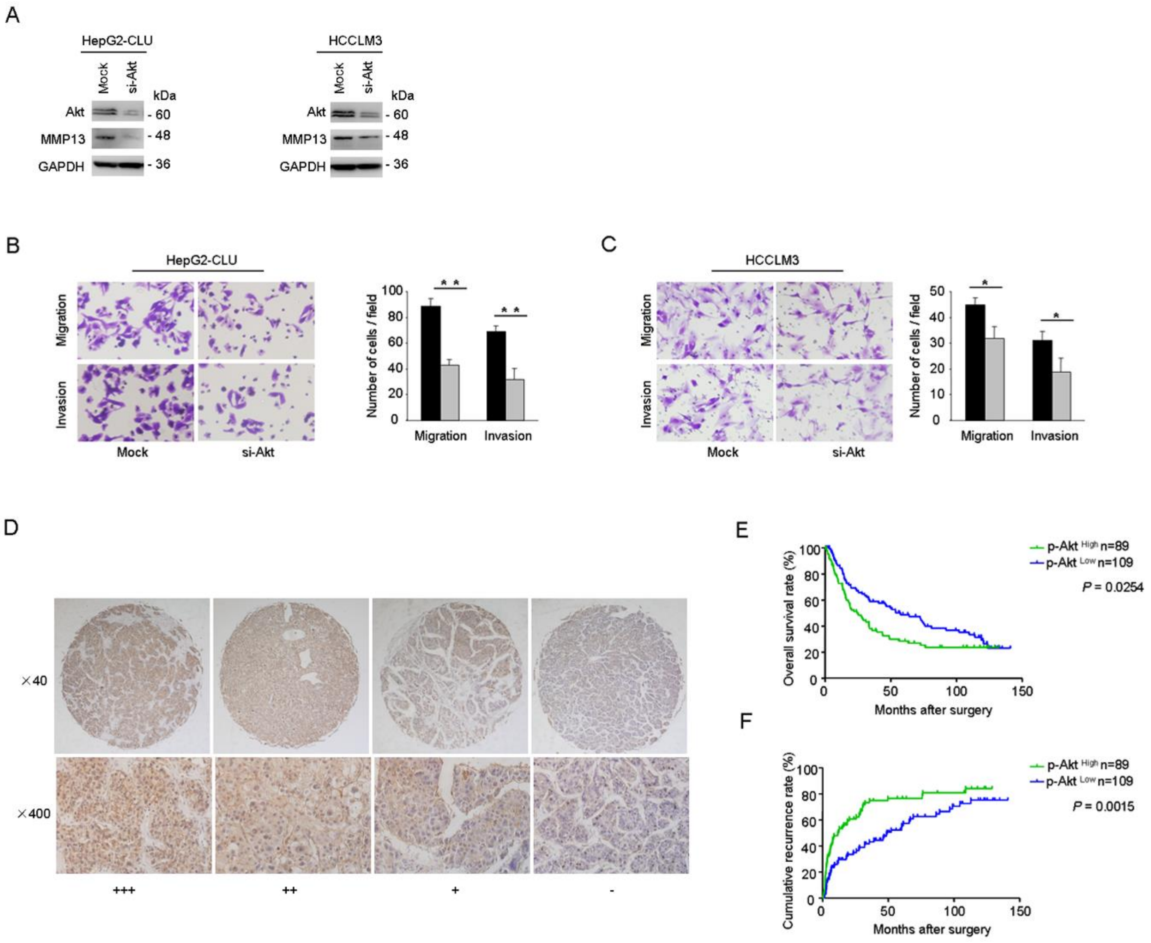

Supplementary Figure 3: Inhibitory effect of Akt siRNA on migration and invasion of HepG2-CLU and HCCLM3 cells.

(A) HepG2-CLU and HCCLM3 cells were treated with Akt siRNA. Levels of Akt and MMP13 were detected by western blotting. (B and C) Migration and matrigel invasion assays were done for the HepG2-CLU and HCCLM3 cells following treatment with Akt siRNA (magnification, ×400). Each experiment was performed in triplicate. (D) Representative images of p-Akt in HCC tissue microarrays by immunochemistry analysis (score +++: strong positive; score ++: moderate positive; score +: weak positive; score -: negative); (E and F) Kaplan-Meier analysis of OS and TTR in 198 HCC cases based on

p-Akt level. *P < 0.05, **P < 0.01.

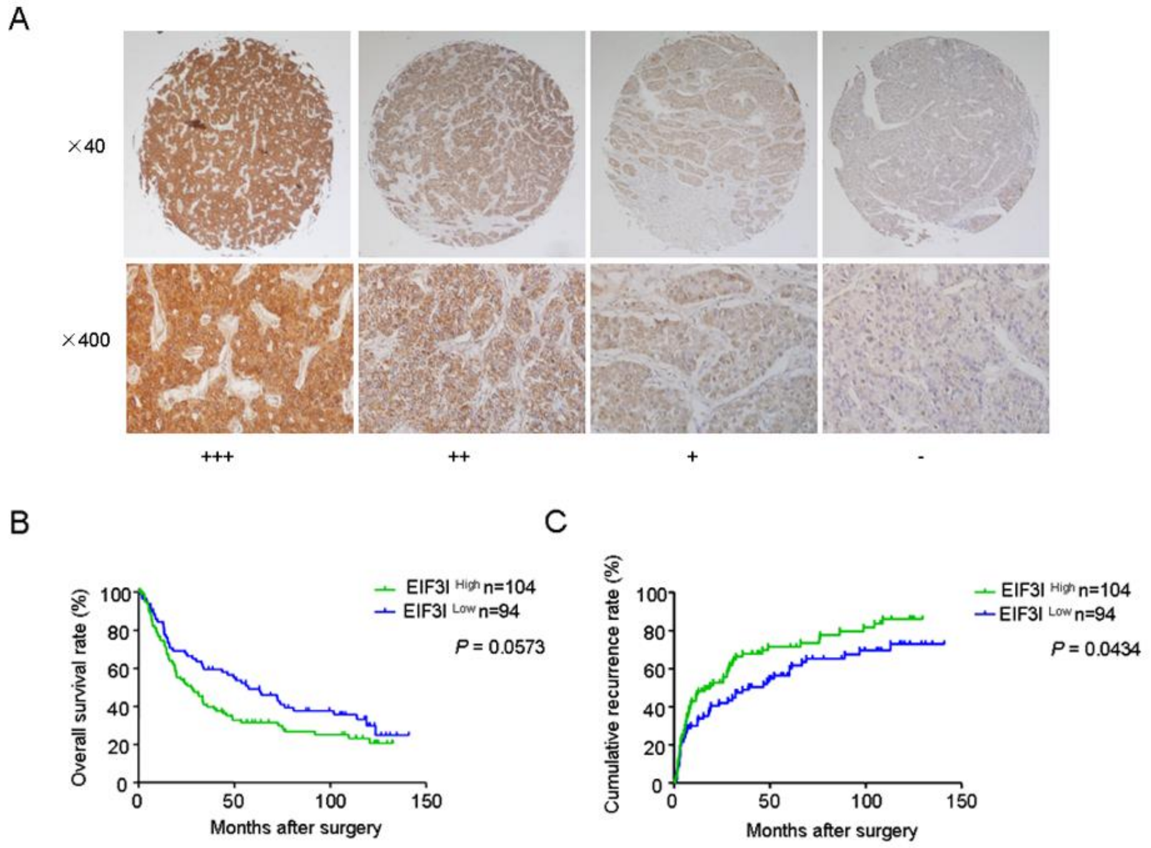

Supplementary Figure 4: Predictive significance of EIF3I expression on survival and recurrence in HCC patients.

(A) Typical expressions of EIF3I in HCC tissue microarrays by immunochemistry analysis (score +++: strong positive; score ++: moderate positive; score +: weak positive; score -: negative). (B and C) Kaplan-Meier analysis of OS and TTR in 198 HCC cases based on EIF3I expression.

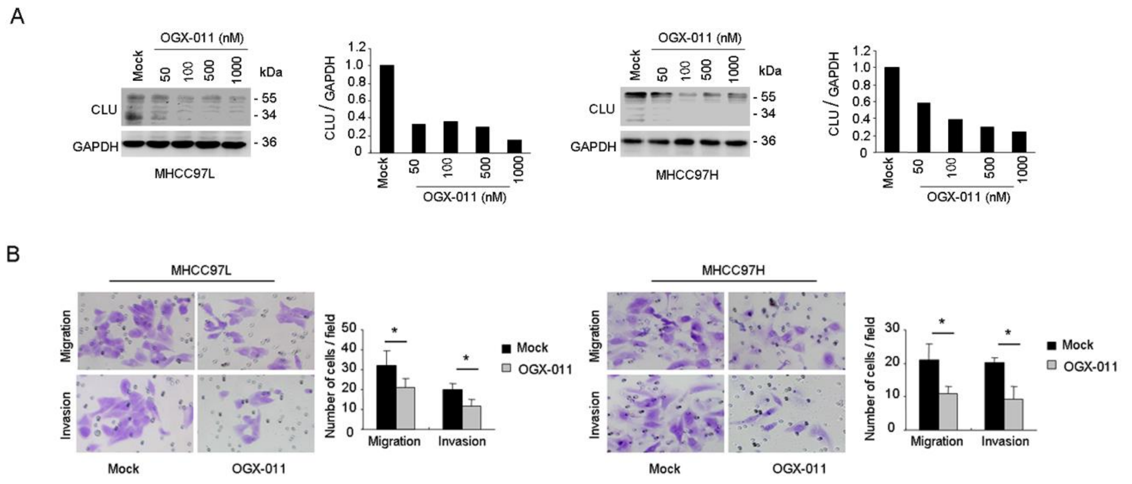

Supplementary Figure 5: OGX-011 significantly suppresses HCC migration and invasion *in vitro.*

(A) MHCC97L and MHCC97H cells were treated with different concentration of OGX-011 for 48h. Protein extracts were analyzed for CLU and GAPDH. (B and C) Migration and matrigel invasion assays showed that OGX-011 significantly suppressed migrative and invasive ability of MHCC97L and MHCC97H cells (magnification, ×400). Each experiment was performed in triplicate. *$P < 0.05$.
